# Low-Cost, Reusable Fracture Reduction Task Trainer for Distal Radius Fractures

**DOI:** 10.5070/M5.52357

**Published:** 2026-04-30

**Authors:** Gabriela Guez, Stephanie Stapleton

**Affiliations:** * Boston Medical Center, Department of Emergency Medicine, Boston, MA

## Abstract

**Audience:**

This task trainer is designed to instruct emergency medicine (EM) resident physicians, senior medical students, and advanced practice providers.

**Introduction:**

While orthopedic emergencies are common, exposure to fracture management may vary depending on one’s training environment. Given that extremity bony trauma and fracture reduction techniques are listed as core content topics in the American Board of Emergency Medicine’s (ABEM) 2022 Model of the Clinical Practice of Emergency Medicine,^1^ it is beneficial to practice their management in a simulated environment. Fracture reduction is particularly difficult to practice without any task trainers. Commercial fracture models are available and cost thousands of dollars which limits universal availability. This wrist fracture task trainer was developed to be a low-cost, reusable, transportable, and storable model with similar haptics to human bone and soft tissue.

**Educational Objectives:**

Utilizing this task trainer, learners will be able to 1) identify key anatomic structures, 2) distinguish a Colles from a Smith fracture of the radius, 3) understand fracture reduction technique using traction, translation and angulation, 4) appreciate the amount of force required for manipulation of the distal fracture fragment, and 5) gain hands-on practice using a model with similar haptics to bone and soft tissue.

**Educational Methods:**

This task trainer is designed for practicing fracture reduction techniques in a simulated environment. Using materials that mimic similar haptics to bone and soft tissue, such as taut rubber bands for tendons and layers of socks for soft tissue, learners will need to use force to apply traction and reduce the distal fracture fragment through translation and angulation. This task trainer can be used for procedural education at the bedside or as part of small group sessions.

**Research Methods:**

This task trainer was used during a small group session at a training institution’s medical simulation conference. Using a Likert scale, an anonymous survey assessed learner’s confidence in the ability to reduce a fracture pre- and post-session. Free-text boxes were also provided to obtain feedback. This task trainer was also presented as a table-top innovation in an EM national conference where attendees provided verbal feedback.

**Results:**

This task trainer was utilized by 32 participants during a medical simulation conference (10 PGY-1 (Post Graduate Year-1), 7 PGY-2, 5 PGY-3, 3 PGY-4, 5 medical students, 1 fellow, 1 attending physician). Of these individuals, 23 participated in the anonymous survey (8 PGY-1, 6 PGY-2, 4 PGY-3, 3 PGY-4, 2 medical students). Using a Likert scale of 1 to 5 assessing learner’s confidence in fracture reduction technique before and after using this task trainer, the total mean score of the pre-session survey was 2.6 and the total mean score of the post-session survey results was 3.9. Attendees of an EM national conference who used the task trainer for hands-on practice provided verbal feedback. It was stated that its design was simple enough to replicate, it could be used for bedside teaching during a shift, and that the feel of the fracture line and technique required to reduce the fragment was realistic. It was noted that although the amount of force required was less for this model than in human practice, it was conceptually accurate.

**Discussion:**

The utilization of this task trainer allows learners to understand and practice the maneuvers and force needed for successful fracture reduction. The materials used in its design require the learner to manipulate the distal fracture fragment in a realistic manner. This model is a low cost, reusable, transportable, and easily storable alternative to commercial trainers and can be used in a simulated environment to improve procedural competency and confidence.

**Topics:**

Fracture reduction, orthopedic emergencies, distal wrist fracture, distal radius fracture, Colles fracture, Smith fracture, radius, ulna.

## USER GUIDE

**Table:** 

List of Resources:	
Abstract	34
User Guide	35
Appendix A: Teaching Pearls	41

**Learner Audience:**

Senior Medical Students, Interns, Junior Residents, Senior Residents, EM Advanced Practice Providers

**Time Required for Implementation:**

Once supplies are obtained, it should take the instructor less than 30 minutes to create the model. Each learner will use the task trainer for approximately 5 minutes.

**Recommended Number of Learners per Instructor:**

Ratio of 1 instructor to 5 learners maximum.

**Topics:**

Fracture reduction, orthopedic emergencies, distal wrist fracture, distal radius fracture, Colles fracture, Smith fracture, radius, ulna.

**Objectives:**

Utilizing this task trainer, learners will be able to:

1. Identify key anatomic structures
2. Distinguish a Colles from a Smith fracture of the radius
3. Understand fracture reduction technique using traction, translation and angulation
4. Appreciate the amount of force required for manipulation of the distal fracture fragment
5. Gain hands-on practice using a model with similar haptics to bone and soft tissue

### Linked objectives and methods

The utilization of this task trainer allows learners to understand the technique of fracture reduction (Objective 3), appreciate the amount of force required for manipulation (Objective 4), and gain hands-on procedure practice (Objective 5). Especially in training environments with infrequent exposure to fracture reduction, using this task trainer in simulated settings can improve skill competency and confidence. While commercial models can be cost prohibitive, this designed innovation is low-cost, reusable, and easy to transport and store.

Although the skeletal bones of the task trainer are covered in socks and placed in a long cloth glove, they can be easily removed from these layers to facilitate the discussion of basic anatomy (Objective 1) and the distinguishing features of Colles and Smith fractures (Objective 2). The taut rubber bands that span across the fracture fragment mimic tendons that require the learner to use an appropriate amount of force to maneuver the bones for successful reduction. The layers of socks and the glove mimic the haptics of soft tissue when feeling for the fracture line and provide better visual fidelity.

### Recommended pre-reading for instructor

Review the provided handout of teaching pearls to facilitate the instruction and discussion of the educational objectives. The following online educational materials are also recommended for pre-session review:

- Overview of radius fractures (*John Hopkins Medicine*): https://www.hopkinsmedicine.org/health/conditions-and-diseases/distal-radius-fracture-wrist-fracture^2^
- Overview of radius fractures, management, and complications (*Orthobullets*): https://www.orthobullets.com/trauma/1027/distal-radius-fractures^3^
- Reduction and splinting of distal radius fractures (*ORTHOfilms*): https://www.youtube.com/watch?app=desktop&v=cy6f7he2e4w^4^
- Hematoma block and Colles fracture reduction (*YouTube*): https://www.youtube.com/watch?app=desktop&v=EhJ7kpurKnk&embeds_referring_euri=https%3A%2F%2Fcoreem.net%2F&source_ve_path=MTM5MTE3LDI4NjY2&feature=emb_logo^5^

Optional for review:

- Splinting guide (*Provider Practice Essentials*): https://ppemedical.com/blog/splinting-guide^6^
- Overview of hematoma blocks (*WikiSM*): https://wikism.org/Hematoma_Block^7^
- Procedural video of a hematoma block of the wrist (*Brown Emergency Medicine Blog*): https://brownemblog.com/blogposts/2021/4/22/hematoma-block-of-the-wrist^8^

### Implementation Methods

This task trainer can be used in a small group session as part of a larger workshop or for bedside teaching. For our purposes, it was used during one of several stations during a medical simulation conference at our training institution practicing the management of various orthopedic emergencies. Prior to learners’ practice with the task trainer, the instructor should verbally review basic anatomy, highlight the differences between Colles and Smith fractures, discuss mechanisms of injury, and consider the potential injuries associated with these fractures. Optional topics for discussion can include describing the procedural steps of a hematoma block for analgesia prior to reduction and post-reduction splinting methods.

Using the distal wrist fracture task trainer, the instructor should walk the learners through the techniques of fracture reduction and emphasize the amount of force that would realistically need to be used. While the task trainer is designed to have skeletal bones placed inside layers of socks and in a long cloth glove for better haptics and visual fidelity, the bones can be easily removed from these layers to facilitate the discussion of anatomy and reduction maneuvers. The learners will practice applying traction to the distal fracture fragment and then translating and angulating the bones for successful reduction. The learners can practice as many times as they need to improve their procedural competency and confidence.

### List of items required to replicate this innovation

Items purchased online on Amazon:

1. Anatomically accurate replica of a Radius
2. Anatomically accurate replica of an Ulna
3. Several thick rubber bands (7'' x 5/8'') with loops cut once to make flat strips
4. Several hair ties (2 mm were used)
5. Long cloth gardening glove

The following items and materials were used from home:

1. Small hand saw or Dremel rotary tool
2. Several pairs of socks
3. Tissue paper or gauze roll

### Approximate cost of items to create this innovation

Approximately $50 US dollars at the time of online purchase on Amazon.

### Detailed methods to construct this innovation

1. Use a small hand saw or Dremel rotary tool to cut the radial bone at the distal end to create the fracture fragment. It is best to have an approximately 2-inch distal fracture fragment for ease of design and practice.[Fig f1-jetem-11-2-i34]
2. Place two rubber band strips axially along the radial bone, one on each side of the bone, spanning across the fracture site to mimic tendons.[Fig f2-jetem-11-2-i34]
3. Tightly secure the rubber bands to the radius with a hair tie proximal to the fracture line.[Fig f3-jetem-11-2-i34]
4. Tightly secure the rubber bands with a second hair tie distal to the fracture line.[Fig f4-jetem-11-2-i34]
5. Pull on the ends of the rubber band strips in opposite directions to tighten them against the bone. The goal is to make the rubber bands very taut so that force is needed to overcome their slack when the fracture fragment is displaced.[Fig f5-jetem-11-2-i34][Fig f6-jetem-11-2-i34][Fig f7-jetem-11-2-i34]
6. Given that the radius and ulna are purchased as disarticulated bones, use hair ties and/or rubber bands to affix them to each other at their proximal ends. Of note, extra rubber band strips may need to be wedged in between the two bones to create space between them and keep them aligned.[Fig f8-jetem-11-2-i34]
7. Cover the entire skeletal model with 4–6 layers of socks, layered flat one on top of another, to replicate soft tissue.[Fig f9-jetem-11-2-i34]
8. Stuff each finger of a cloth glove with tissue paper or gauze to give it a realistic appearance of a hand.[Fig f10-jetem-11-2-i34]
9. Place the model inside the long cloth glove for better visual fidelity of an upper extremity.[Fig f11-jetem-11-2-i34]

### Results and tips for successful implementation

This task trainer can be used as either a bedside teaching tool to review procedural technique prior to performance on a patient or in a small group session as part of a larger educational meeting which may allow for broader discussions regarding fracture reduction. Our task trainer was used during a session as part of a larger medical simulation conference for orthopedic emergencies held for residents and medical students of a single academic institution. After the sessions, participants were provided an anonymous survey assessing their confidence in the ability to reduce a fracture pre- and post-session using a Likert scale from 1–5. A score of 1= Not Confident, and a score of 5= Very Confident. Free-text boxes were also provided for feedback. Study data were collected and managed using REDCap electronic data capture tools hosted at Boston University Clinical & Translational Science Institute grant 1UL1TR001430 and analyzed via Google Sheets. This task trainer was utilized by 32 participants (10 PGY-1, 7 PGY-2, 5 PGY-3, 3 PGY-4, 5 medical students, 1 fellow, 1 attending physician) of which 23 individuals (8 PGY-1, 6 PGY-2, 4 PGY-3, 3 PGY-4, 2 medical students) participated in the anonymous survey. In assessing their level of confidence in fracture reduction techniques before and after using this task trainer, the total mean score of the pre-session survey was 2.6 and the total mean score of the post-session survey results was 3.9. Written feedback was positive overall with participants expressing appreciation for the opportunity to discuss reduction techniques and practice the procedure. This task trainer was also presented as a table-top innovation at a national EM conference where attendees could use the model for hands-on practice. Verbal feedback included that its design was easily reproducible, it could be used for bedside teaching in the emergency department, and that the feel of the fracture line and technique required for reduction was realistic. It was mentioned, however, that the amount of force required to reduce the fracture was less accurate than in human practice. After having been used by several learners during small group sessions and attendees of the national conference, it is suggested that the integrity of the model be checked every few participants to make sure the rubber band strips have remained taut and in place.

### Associated content

- The teaching pearls handout is intended to be reviewed by the instructor prior to the session but may also serve as a reference during the activity.

## Figures and Tables

**Figure 1 f1-jetem-11-2-i34:**
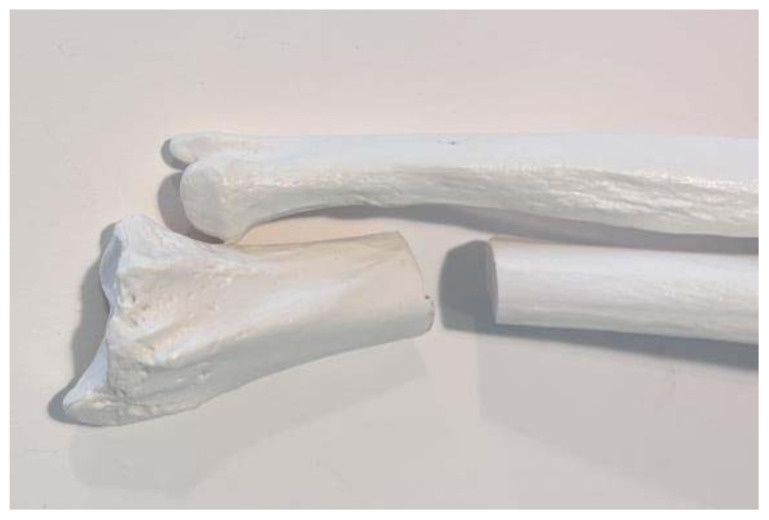
Radial bone cut at approximately 2 inches from the distal end: Author's own image

**Figure 2 f2-jetem-11-2-i34:**
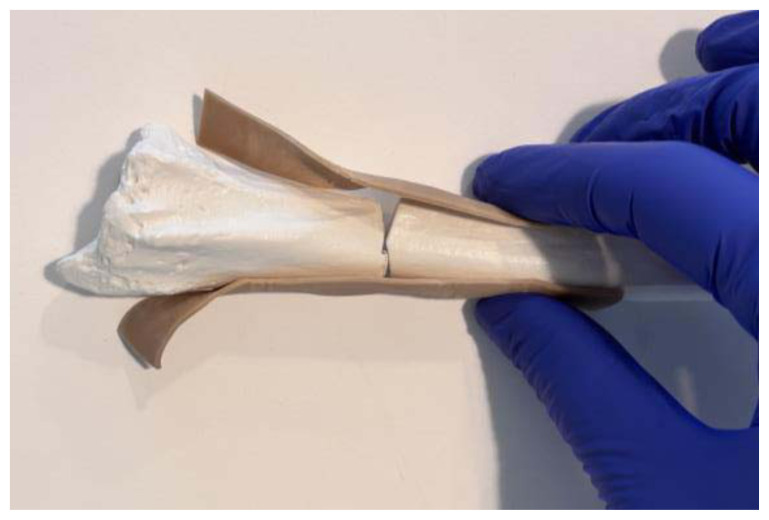
Rubber band strips placed axially along the radius, one on each side, spanning the fracture site: Author’s own image.

**Figure 3 f3-jetem-11-2-i34:**
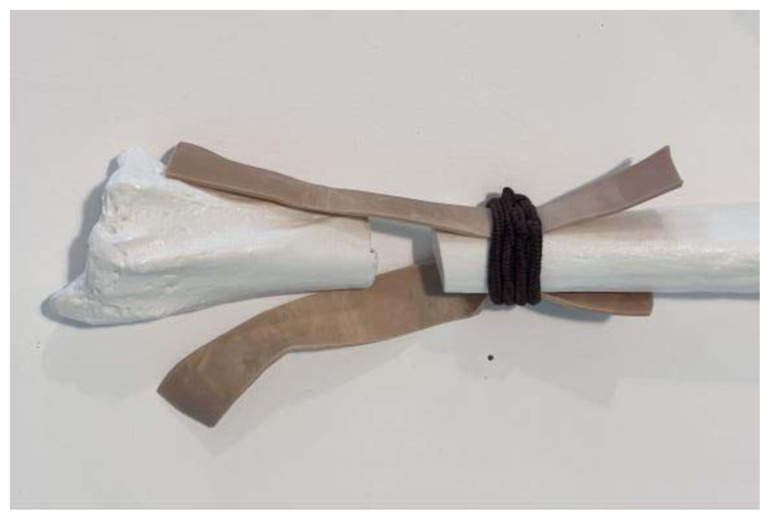
A hair tie securing the rubber bands proximal to the fracture line: Author’s own image.

**Figure 4 f4-jetem-11-2-i34:**
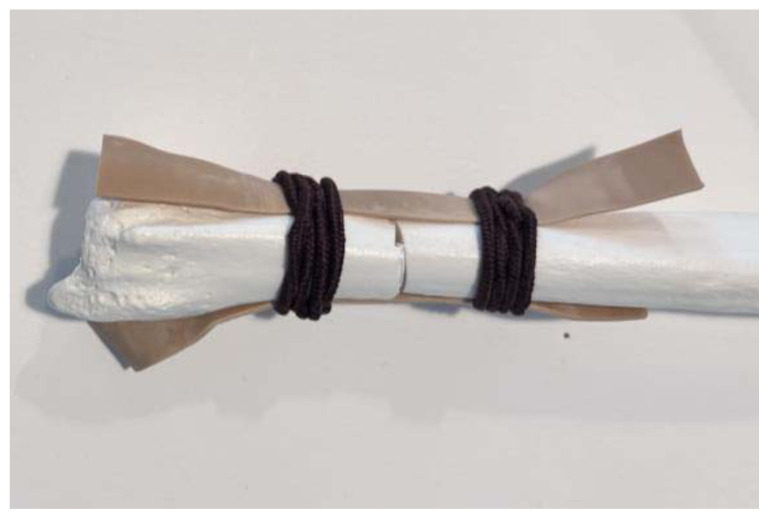
A hair tie securing the rubber bands distal to the fracture line: Author’s own image.

**Figure 5 f5-jetem-11-2-i34:**
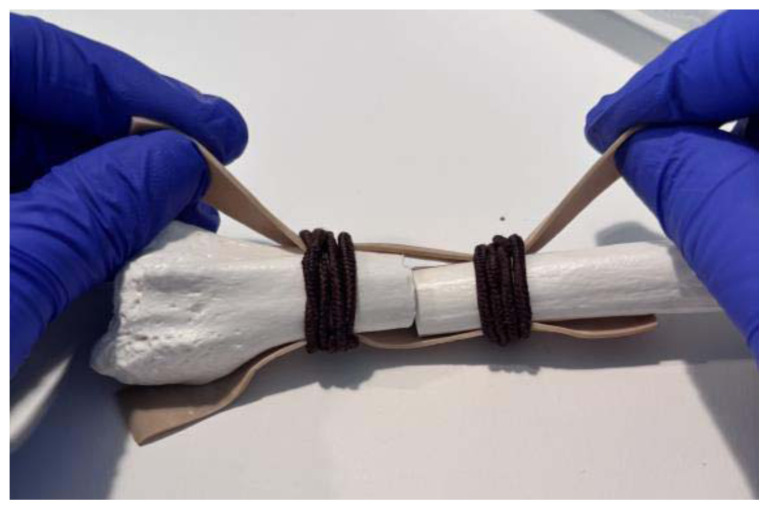
Pull the ends of the rubber bands in opposite directions to make them tight against the bone: Author’s own image.

**Figure 6 f6-jetem-11-2-i34:**
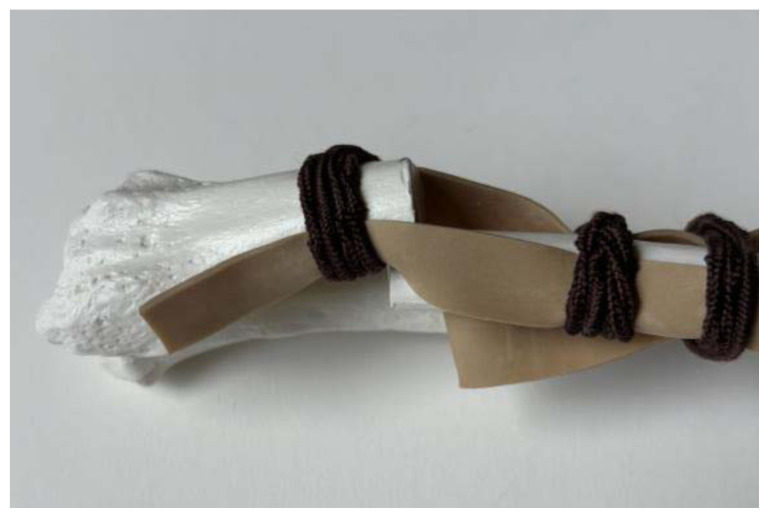
Slack rubber bands when fracture is displaced: Author’s own image.

**Figure 7 f7-jetem-11-2-i34:**
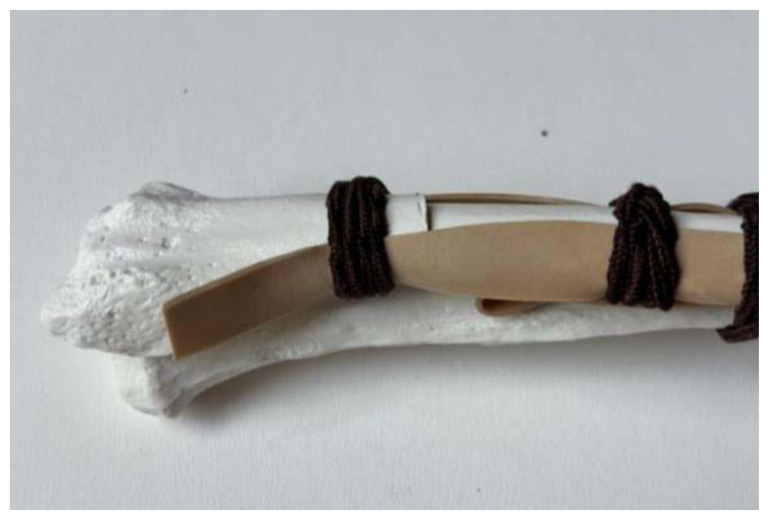
Taut rubber bands when fracture is reduced: Author’s own image.

**Figure 8 f8-jetem-11-2-i34:**
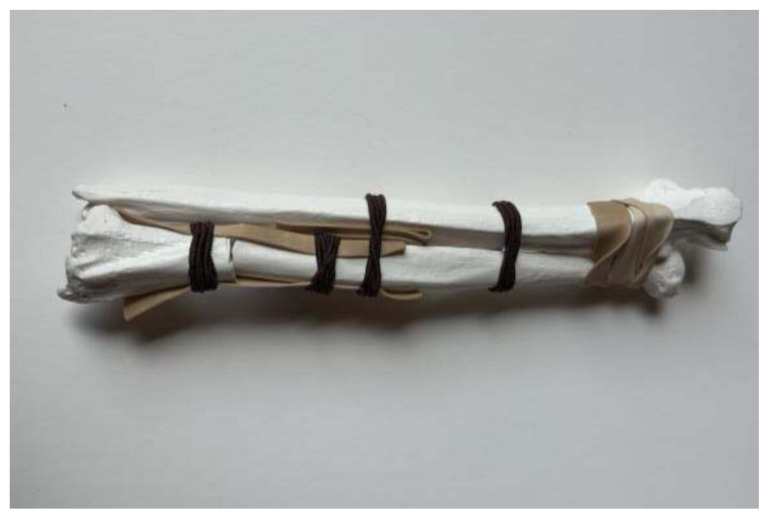
Radius and Ulna affixed with hair ties and/or rubber bands: Author’s own image.

**Figure 9 f9-jetem-11-2-i34:**
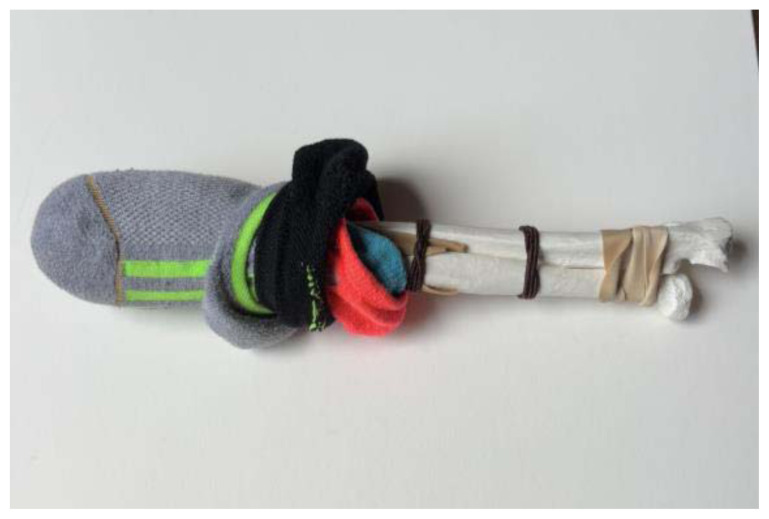
Layers of socks placed over skeletal model to replicate soft tissue. In this image, socks are rolled back to demonstrate the layered configuration: Author’s own image.

**Figure 10 f10-jetem-11-2-i34:**
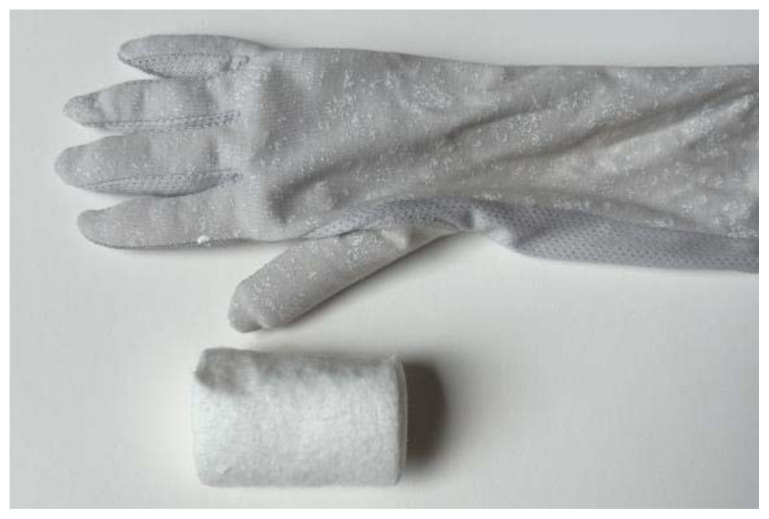
Fingers of a cloth glove stuffed to give a realistic appearance of a hand.

**Figure 11 f11-jetem-11-2-i34:**
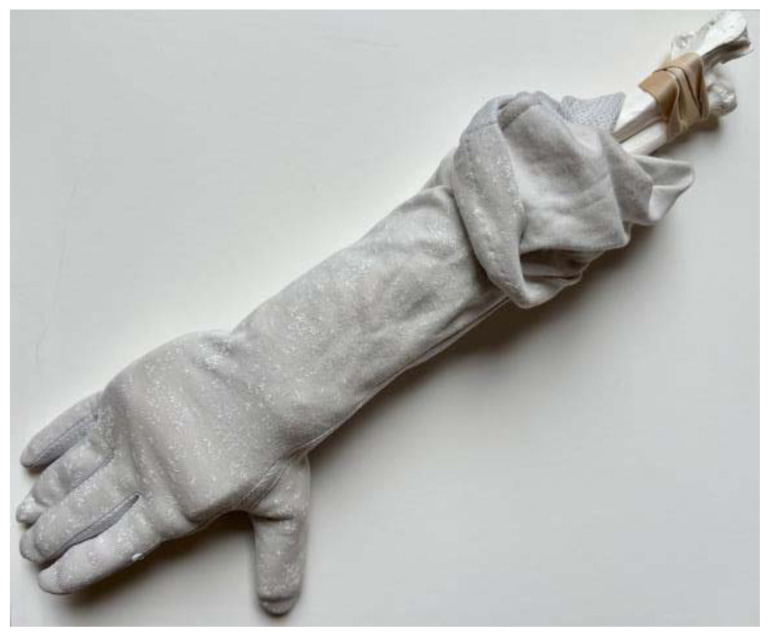
Entire model placed inside a long cloth glove for visual fidelity of an upper extremity.

## INSTRUCTOR MATERIALS

### Appendix A Teaching Pearls

#### Distal Wrist Fractures

- Basic anatomy:^2^
  ○ Radius- forearm bone on the thumb side
  ○ Ulna- forearm bone on the pinky finger side
- Mechanisms of injury:^2^
  ○ Fall on an outstretched hand (FOOSH)
  ○ Fall on a flexed hand
  ○ Can occur from a standing-height fall in older populations with lower bone density or from higher injury mechanisms (motor vehicle collisions and sports) in younger populations
- Colles vs Smith fracture classification:^2^
  ○ Colles: The broken fragment of the distal radius bone is dorsally angulated. This creates a “dinner fork deformity” where there is a bump at the wrist, like the neck of a fork. This injury usually results from impact to the palm in a FOOSH mechanism.
  ○ Smith: The broken fragment of the distal radius bone is angled toward the palm. This creates an appearance of a distinct drop in the wrist. This injury usually results from impact to the back of the wrist, likely falling on a bent/flexed wrist.
- More common potential injuries associated with fracture:^3^
  ○ Distal radioulnar joint injury
  ○ Median nerve neuropathy (carpal tunnel syndrome)
  ○ Ulnar nerve neuropathy
  ○ Radiocarpal arthrosis
  ○ Compartment syndrome
- Basic technique of fracture reduction:^4^,^5^
  ○ Operator uses both their hands to encircle the affected distal forearm in a “C”-shape, with fingers on ventral aspect of the forearm and thumbs on its dorsal aspect with thumbs placed up against the fracture fragment by the fracture line
  ○ Throughout the procedure, longitudinal traction is simultaneously being applied to the proximal forearm by an assistant or by using traction devices such as finger trap systems.
  ○ Operator re-creates the deformity of the broken bone fragment by angulating it dorsally (Colles) or ventrally (Smith) while applying longitudinal traction to free it from its impaction against the proximal radius.
  ○ Operator then applies volar (Colles) or dorsal (Smith) pressure to the distal fracture fragment to hinge it up and over the proximal radius to align it with the proximal forearm.
  ○ Fracture is reduced when the bones are in appropriate alignment.

#### Optional points for discussion

- Post-reduction management:^6^
  ○ Apply sugar-tong splint to extremity to hold fracture in alignment
  ○ Obtain an x-ray image to confirm fracture reduction
- Hematoma block for pre-reduction anesthesia:^7^,^8^
  ○ Drape and prep the distal radius in sterile fashion. Draw up 5–10 mL of lidocaine into a 10-cc syringe. Attach a 25–27-gauge needle and superficially inject lidocaine to create a small skin wheal. Remove the small gauge needle and attach an 18-gauge needle to the syringe. Insert the needle into the skin directly over the fracture line and aspirate while advancing until you encounter bone. Aspirating blood at the fracture line confirms that the needle tip is within the hematoma. Inject 5–10 mL of lidocaine into the hematoma to bathe the periosteum in anesthetic.
